# Notes and Notices

**Published:** 1896-04

**Authors:** 


### NOTES AND NOTICES.

International Hahnemannian Association.—The annual meeting of the International Hahnemannian Association will be held at Glen Summit, at the Glen Summit Hotel, Luzerne County, Pa., June 24th next.

The chairmen of the bureaus are Geo. H. Clark, M. D., for Homœopathic Philosophy; Arthur G. Allan, M. D., for Materia Medica; Annie L. Geddes, M. D., for Clinics; Howard Crutcher, M. D., for Surgery; Caroline E. Hastings, M. D., for Obstetrics; Stuart Close, M. D., Necrologist.

The titles of papers should be sent to the chairmen no later than May 1st, and the papers as early as May 15th.

E. E. Case, M. D., *Secretary.*

Homœopathic Medical College of Missouri.—The thirty-ninth annual commencement exercises of the Homœopathic Medical College of Missouri were held last night at Pickwick Theatre, Washington Avenue near Jefferson before an audience which completely filled the hall.

On the stage were seated the members of the Board of Trustees of the college, and the ladies and gentlemen who rendered the various musical numbers on the programme. The board is composed of the following: W. A. Edmonds, A. M., M. D.; L. C. McElwee, M. D.; James A. Campbell, M. D.; I. D. Foulon, A. M., M. D., LL. D.; W. C. Richardson, M. D.; A. H. Schott, M. D.; S. B. Parsons, M. D.; J. Martine Kershaw, M. D.; W. B. Morgan, M. D., Ph. D.; W. L. Reed, M. D.; W. A. Wilcox, M. D.

Dr. W. C. Richardson is Dean of the institution, and to him was assigned the duty of introducing the participants.

Nineteen ladies and gentlemen compose this year's graduating class of the college. They are: C. R. Armstrong, Samuel A. Benson, J. W. Koelle, L. Herckenroeder, W. J. Morris, Edward Ellerbrock, Jennie Walker, Fred Gessner, W. E. Rieley, A. LeRoy Barnard, H. S. Bauer, Maud G. Keller, H. D. McKenzie, E. W. Burkhardt, Frank Zillicken, G. A. Paulisch, W. A. Carriere, F. W. Murck, H. G. Isenberg.

Rev. Dr. M. G. Gorin pronounced the invocation, and W. A. Edmonds, A. M., M. D., conferred the degree of Doctor of Medicine upon each of the graduates, at the same time making a few remarks appropriate to the occasion. The address of the evening was delivered by Rev. Michael Burnham, D. D., pastor of the Pilgrim Congregational Church. Dr. Burnham congratulated the graduates upon successfully passing the trying ordeal of a final examination. He commented on the innumerable instances which present themselves to physicians to help in some manner or other the unfortunate and helpless, and said he hoped the class of '96 would never neglect an opportunity of doing God's work. His remarks were followed attentively by the students, and the Reverend Doctor was enthusiastically applauded when he closed.

The presentation of the class honors was made by Dr. I. D. Foulon, after which the bouquets were distributed among the graduates. As each stepped up to receive the testimonial of his or her friends, the audience showed its appreciation by hearty applause. This was particularly true in the case of the two ladies who were members of this year's graduating class.—*St. Louis Daily Globe Democrat*, April 3d, 1896.

Annual Banquet—It is Enjoyed by the Alumni of the Homœopathic Medical College.—Fully two hundred and fifty persons attended the annual banquet of the alumni of the Homœopathic Medical College of Missouri at the Southern Hotel, April 2d. The spread was excellent and the toasts equally good. Frederick H. Bacon officiated as toastmaster.

Dr. Wm. C. Richardson responded to the toast "Alma Mater." Dr. John W. Streeter spoke to "The Homœopathic Physician—the Modern Magican," while the toast "Humanity's Need—A Free Homœopathic Hospital," was responded to by Rev. Dr. George Edward Martin.

The remainder of the toasts and responses were as follows: "Woman in Medicine—She Has Come to Stay," by Dr. Alice Butterworth; "The Doctor and the Lawyer—Friends and Allies," Mr. Ford Smith; "The Ladies," Rev. Dr. J. Henry George; "The Young Physician—Large in Ambition Though Small in Purse," Mr. George A. Lemming.

Dr. Amon T. Noe has removed from St. Louis to Bement, Ill.

"A Bright Lad That Was."—A teacher told the pupils to make up a sentence or "story" from the suggestive words, "boys," "bees," "bear." Quick as flash up come one hand, "I have it." "What is it, Tommy?" inquired the teacher. "Boys bees bare when they go in swimming," was the astonishing reply. A better sentence would have been—"*Boys* will be interested in *bees* and other insects, *bears* and other animals as well as birds, flowers, etc., as described in *The Observer*, Portland, Conn. Sample, 10 cents. One year, $1.00.

Annual Re-union of the Alumni Association of the Hahnemann Medical College, Philadelphia, Tuesday, May 5th, 1896.—The Annual Re-union and Banquet of the Alumni Association of the Hahnemann Medical College, Philadelphia, will be held on Tuesday, May 5th, 1896.

The Business Meeting will convene at 4.30 P. M. in Alumni Hall, Hahnemann Medical College, Broad Street above Race, Philadelphia, and the Banquet will be held at 9.45 P. M. at the "Walton," S. E. Corner of Broad and Locust Streets.

The Trustees and Faculty of the College extend a cordial invitation to all the members of the Alumni and their friends to attend the Forty-eighth Annual Commencement, to be held on the same evening, at 8 o'clock, at the Academy of Music, S. W. Corner Broad and Locust Streets, Philadelphia.

Banquet cards can be secured by notifying the Secretary. Requests received after Saturday, May 2d, 1896, cannot be considered.

W. W. Van Baun, M. D., *Secretary*,
419 Pine Street, Philadelphia.

The American Medical Association meets in Atlanta, Ga., May 5th, and all delegates to this Society should embrace the opportunity of visiting "Lookout Inn," a magnificent and historic spot on the crest of Lookout Mountain, overlooking Chattanooga, Tenn. A special invitation is extended to physicians to spend a week with us, en route to Atlanta, and a complimentary rate will be made for the occasion. Trains up the mountain make close connections in both the Central and Union Depots, Chattanooga, running through to the Inn without change. For further information and handsome, illustrated booklet, address M. S. Gibson, Lookout Mountain, Tennessee.

